# Blood transfusion risk prediction in spinal tuberculosis surgery: development and assessment of a novel predictive nomogram

**DOI:** 10.1186/s12891-022-05132-z

**Published:** 2022-02-25

**Authors:** Liyi Chen, Zhaoping Gan, Shengsheng Huang, Tuo Liang, Xuhua Sun, Ming Yi, Shaofeng Wu, Binguang Fan, Jiarui Chen, Tianyou Chen, Zhen Ye, Wuhua Chen, Hao Li, Jie Jiang, Hao Guo, Yuanlin Yao, Shian Liao, Chaojie Yu, Chong Liu, Xinli Zhan

**Affiliations:** 1 Spine and osteopathy ward, First Affiliated Hospital of GuangXi Medical University, Nanning, Guangxi Province China; 2 Department of Hematology, First Affiliated Hospital of GuangXi Medical University, Nanning, Guangxi Province China

**Keywords:** Transfusion, Spinal tuberculosis, Nomogram, Surgery

## Abstract

**Objective:**

The present study attempted to predict blood transfusion risk in spinal tuberculosis surgery by using a novel predictive nomogram.

**Methods:**

The study was conducted on the clinical data of 495 patients (167 patients in the transfusion group and 328 patients in the non-transfusion group) who underwent spinal tuberculosis surgery in our hospital from June 2012 to June 2021. The least absolute shrinkage and selection operator (LASSO) and multivariable logistic regression analyses were used to screen out statistically significant parameters, which were included to establish a novel predictive nomogram model. The receiver operating characteristic (ROC) curve, calibration curves, C-index, and decision curve analysis (DCA) were used to evaluate the model. Finally, the nomogram was further assessed through internal validation.

**Results:**

The C-index of the nomogram was 0.787 (95% confidence interval: 74.6%–.82.8%). The C-value calculated by internal validation was 0.763. The area under the curve (AUC) of the predictive nomogram was 0.785, and the DCA was 0.01–0.79.

**Conclusion:**

A nomogram with high accuracy, clinical validity, and reliability was established to predict blood transfusion risk in spinal tuberculosis surgery. Surgeons must prepare preoperative surgical strategies and ensure adequate availability of blood before surgery.

## Introduction

Spinal tuberculosis is the most common extrapulmonary tuberculosis [1] and is a major global healthcare challenge, particularly in developing countries [2]. Typical clinical manifestations of spinal tuberculosis include pain, fatigue, night sweats, low fever, weight loss, and other symptoms of tuberculosis poisoning [3]. Surgery is required when conservative treatment for spinal tuberculosis fails due to drug resistance [4, 5]. Surgical approaches include anterior, posterior, and combined anteroposterior approaches [6, 7]. Regardless of the surgical approach, preoperative blood preparation is essential for the perioperative period [8].

However, blood resources were very rare, and the problem of blood shortage is common. Blood products cannot meet the growing surgical demand [9]. The surgical procedure for spinal tuberculosis is difficult, and severe intraoperative bleeding occurs during surgery [10]. Shi et al. demonstrated a bleeding volume of 350–550 mL for spinal tuberculosis surgery [5], whereas Li et al. exhibited a volume of > 500 mL [3]. The delay in surgery due to inadequate preoperative blood preparation is deleterious for patients. The requirement for blood transfusion in spinal tuberculosis surgery depends on several factors such as anaemia caused due to a deficiency of iron [11], vitamin B12, and folic acid [12], which are used to compound haemoglobin (HGB) and sex of the patient [13–15]. Dong et al. exhibited that the factors such as preoperative mean corpuscular haemoglobin concentration, surgical duration, preoperative HGB, intraoperative blood loss, number of fused vertebrae, and anticoagulant history can predict the risk of blood transfusion after spinal tuberculosis fusion [16].

Although several factors affect blood transfusion in spinal tuberculosis surgery, no consensus is available on the factors that predict intraoperative blood transfusion in the perioperative period. The present study reports a novel predictive nomogram to predict blood transfusion risk in spinal tuberculosis surgery, which is crucial for the reasonable and scientific management of blood transfusion [17].

## Methods

### Patients

The present study was conducted on the clinical data of 495 patients who underwent spinal tuberculosis surgery in the First Affiliated Hospital of Guangxi Medical University from June 2012 to June 2021 after institutional ethics clearance. The data of patients with complete perioperative data diagnosed pathologically with spinal tuberculosis and who underwent surgical treatment were included in the study. The criteria for exclusion of data were as follows: patients with incomplete perioperative data in whom the pathological examination ruled out spinal tuberculosis; those with conditions that affected blood transfusion such as bleeding and clotting disorders; those who took drugs that affected their clotting function for nearly a year; and those undergoing conservative treatment.

### Data collection

Perioperative data such as general patient information, clinical manifestations, surgical strategies, and preoperative blood tests were collected. The transformation of perioperative variables into binary variables was based on previous literature reports [18–20]. The presence or absence of pain was based on whether the patient took analgesics. The patients were divided into two groups, namely the transfusion group and non-transfusion group. The scores were determined by averaging the scores of two senior physicians from our department. The imaging evaluation was jointly determined by two senior doctors from the imaging department of our hospital.

### Statistical analysis

Data were analysed using R software for Windows (Version 4.1.0) and auxiliary RStudio software (Version 1.4.1717). All perioperative data were analysed using the LASSO method. Statistically significant parameters were included in multivariable logistic regression analysis after LASSO regression model analysis. Statistically significant parameters from multivariable logistic regression analysis were included in the follow-up analysis. Therefore, a predictive nomogram model was developed. The prediction accuracy and discriminant ability of the nomograms were assessed by the C-index. The range of the C-index was usually < 0.5, 0.5–0.7, 0.7–0.9, and > 0.9, representing low accuracy, moderate accuracy, high accuracy, and extreme accuracy, respectively [21]. A calibration curve was plotted to assess the actual and predicted risks of the blood transfusion nomogram. The AUC was used to evaluate the predictive ability of the nomogram, whereas the DCA curve was used to evaluate the clinical usefulness of the nomogram by quantifying the net benefits. Finally, the internal validation method was used to verify the nomogram. A relatively corrected C-index was calculated by bootstrapping validation (1000 bootstrap resamples) of the blood transfusion nomogram.

## Results

### Patient characteristics

Of the total, 297 patients were men and 198 patients were women. Additionally, 167 patients underwent surgical treatment with blood transfusion, whereas 328 patients underwent surgical treatment without blood transfusion. The perioperative data of the two groups such as general patient information, clinical manifestations, surgical strategies, and preoperative blood tests are presented in Table 1.

**Table 1:** Demographic characteristics of the transfusion and non-transfusion groups

Demographic characteristics	Transfusion group(*n* = 167)	Non-transfusion group(*n* = 328)	Total(*n* = 495)
Gender^a^			
Male	80 (47.90%)	217 (66.16%)	297 (60.00%)
Female	87 (52.10%)	111 (33.84%)	198 (40.00%)
Course of disease(month)^a^			
≦6	106 (63.47%)	238 (72.56%)	344 (69.49%)
> 6	61 (36.53%)	90 (27.44%)	151 (30.51%)
marriage			
Yes	145 (86.83%)	272 (82.93%)	417 (84.24%)
No	22 (13.17%)	56 (17.07%)	78 (15.76%)
Systolic blood pressure(mmHg)			
≦120	62 (37.13%)	136 (41.46%)	198 (40.00%)
> 120	105 (62.87%)	192 (58.54%)	297 (60.00%)
Diastolic blood pressure(mmHg)			
≦80	95 (56.89%)	192 (58.54%)	287 (57.98%)
> 80	72 (43.11%)	136 (41.46%)	208 (42.02%)
BMI(kg/m^2^)^a^			
< 18.5	48 (28.74%)	85 (25.91%)	133 (26.87%)
18.5 ~ 24.9	107 (64.07%)	206 (62.80%)	313 (63.23%)
> 25	12 (7.19%)	37 (11.28%)	49 (9.90%)
Pain^a^			
Yes	164 (98.20%)	305 (92.99%)	469 (94.75%)
No	3 (1.80%)	23 (7.01%)	26 (5.25%)
Lower limb pain			
Yes	95 (56.89%)	170 (51.83%)	265 (53.54%)
No	72 (43.11%)	158 (48.17%)	230,946.46%)
Number of lower limb pain			
0	71 (42.51%)	158 (48.17%)	229 (46.26%)
1	18 (10.78%)	57 (17.38%)	75 (15.15%)
2	78 (46.71%)	113 (34.45%)	191 (38.59%)
Fatigue			
Yes	52 (31.14%)	98 (29.88%)	150 (30.30%)
No	115 (68.86%)	230 (70.12%)	345 (69.70%)
Fever			
Yes	55 (32.93%)	88 (26.83%)	143 (28.89%)
No	112 (67.07%)	240 (73.17%)	352 (71.11%)
Night sweats^a^			
Yes	47 (28.14%)	72 (21.95%)	119 (24.04%)
No	120 (71.86%)	256 (78.05%)	376 (75.96%)
Appetite^a^			
Yes	89 (53.29%)	215 (65.55%)	304 (61.41%)
No	78 (46.71%)	113 (34.45%)	191 (38.59%)
Weight loss			
Yes	73 (43.71%)	117 (35.67%)	190 (38.38%)
No	94 (56.29%)	211 (64.33%)	305 (61.62%)
Surgical approach			
Anterior	99 (59.28%)	190 (57.93%)	289 (58.38%)
Posterior	63 (37.73%)	130 (39.63%)	193 (38.99%)
Anteroposterior	5 (2.99%)	8 (2.44%)	13 (2.63%)
Focal segmental^a^			
≦2	124 (74.25%)	273 (83.23%)	397 (80.20%)
> 3	43 (25.75%)	55 (16.77%)	98 (19.80%)
Intervertebral fusion device			
Yes	82 (49.10%)	147 (44.82%)	229 (46.26%)
No	85 (50.90%)	181 (55.18%)	266 (53.74%)
Internal fixation^a^			
Yes	158 (94.61%)	281 (85.67%)	439 (88.69%)
No	9 (5.39%)	47 (14.33%)	56 (11.31%)
ODI^a^			
≦18	87 (52.10%)	206 (62.80%)	293 (59.19%)
> 18	80 (47.90%)	122 (37.20%)	202 (40.81%)
JOA			
≦20	106 (63.47%)	190 (57.93%)	296 (59.80%)
> 20	61 (36.53%)	138 (42.07%)	199 (40.20%)
VAS			
≦7	83 (49.70%)	167 (50.91%)	250 (50.51%)
> 7	84 (50.30%)	161 (49.09%)	245 (49.49%)
ASIA			
Unimpaired	100 (59.88%)	219 (66.77%)	319 (64.44%)
Impaired	67 (40.12%)	109 (33.23%)	176 (35.56%)
Age(year)^a^			
< 60	85 (50.90%)	236 (71.95%)	321 (64.85%)
≥60	82 (49.10%)	92 (8.05%)	174 (35.15%)
Occupation^a^			
Farmer	109 (65.27%)	191 (58.23%)	300 (60.61%)
Not farmer	58 (34.73%)	137 (41.77%)	195 (39.39%)
Race			
Han	77 (46.11%)	167 (50.91%)	244 (49.29%)
non-Han	90 (53.89%)	161 (49.09%)	251 (50,71%)
Hospitalization days(day)^a^			
≦11	76 (45.51%)	224 (68.29%)	300 (60.61%)
> 11	91 (54.49%)	104 (31.71%)	195 (39.39%)
Surgery classification^a^			
Grade 4	108 (64.67%)	187 (57.01%)	295 (59.60%)
Grade 1–3	59 (35.33%)	141 (42.99%)	200 (40.40%)
Preoperative hospitalization days(day)^a^			
≦4	81 (48.50%)	216 (65.85%)	297 (60.00%)
> 4	86 (51.5%)	112 (34.15%)	198 (40.00%)
Medical insurance			
NCMS	88 (52.69%)	174 (53.05%)	262 (52.93%)
Non- NCMS	79 (47.31%)	154 (46.95%)	233 (47.07%)
Blood glucose(mmol/L)^a^			
3.9 ~ 6.1	138 (82.63%)	252 (76.83%	390 (78.79%)
< 3.9 or > 6.1	29 (17.37%)	76 (23.17%)	105 (21.21%)
Blood type			
A	37 (22.16%)	62 (18.90%)	99 (20.00%)
B	46 (27.54%)	89 (27.113%	135 (27.27%)
AB	7 (4.19%)	24 (7.32%)	31 (6.26%)
O	77 (46.11%)	153 (46.65%)	230 (46.46%)
C-reactive protein(mg/L)			
≦10	65 (38.92%)	148 (45.12%)	213 (43.03%)
> 10	102 (61.08%)	180 (54.88%)	282 (56.79%)
Hepatitis B surface antigen			
Positive	14 (8.38%)	32 (9.76%)	46 (9.29%)
Negative	153 (91.62%)	296 (90.24%)	449 (90.71%)
White blood cells(^a^10^9^/L)^a^			
4 ~ 10	141 (84.43%)	264 (80.49%)	405 (81.82%)
< 4 or > 10	26 (15.57%)	64 (19.51%)	90 (18.18%)
Hemoglobin(g/L)^a^			
< 120	108 (64.67%)	206 (62.80%)	314 (63.43%)
≥120	59 (35.33%)	122 (37.20%)	181 (36.57%)
Platelets(^a^10^9^/L)			
100–300	92 (55.09%)	178 (54.27%)	270 (54.55%)
< 100 or > 300	75 (44.91%)	150 (45.73%)	225 (45.45%)
Percentage of neutrophils(%)			
0.5–0.7	96 (57.49%)	196 (59.76%)	292 (58.99%)
< 0.5 or > 0.7	71 (42.51%)	132 (40.24%)	203 (41.01%)
Percentage of lymphocytes(%)^a^			
0.2–0.4	80 (47.90%)	193 (58.84%)	273 (55.15%)
< 0.2 or > 0.4	87 (52.10%)	135 (41.16%)	222 (44.85%)
Absolute monocytes(^a^10^9^/L)			
0.12–0.8	134 (80.24%)	257 (78.35%)	391 (78.99%)
< 0.12 or > 0.8	33 (19.76%)	71 (21.65%)	104 (21.01%)
Percentage of monocytes(%)			
0.03–0.08	63 (37.72%)	125 (38.11%)	188 (37.98%)
< 0.03 or > 0.08	104 (62.28%)	203 (61.89%)	307 (62.02%)
Total bilirubin(umol/L)			
3.4–17.1	140 (83.83%)	279 (85.06%)	419 (84.65%)
< 3.4 or > 17.1	27 (16.17%)	49 (14.94%)	76 (15.35%)
Direct bilirubin(umol/L)			
≦6.8	149 (89.22%)	306 (93.29%)	455 (91.92%)
> 6.8	18 (10.78%)	22 (6.17%)	40 (8.08%)
Indirect bilirubin(umol/L)			
1.7–10.2	139 (83.23%)	283 (86.28%)	422 (85.25%)
< 1.7 or > 10.2	28 (16.77%)	45 (13.72%)	73 (14.75%)
Total protein(g/L)			
60–80	135 (80.84%)	271 (82.62%)	406 (82.02%)
< 60 or > 80	32 (19.16%)	57 (17.38%)	89 (17.98%)
Albumin(g/L)^a^			
≥40	36 (21.56%)	125 (38.11%)	161 (32.53%)
< 40	131 (78.44%)	203 (61.89%)	334 (67.47%)
Aspartate aminotransferase(U/L)			
10–40	146 (87.43%)	290 (88.41%)	436 (88.08%)
< 10 or > 40	21 (12.57%)	38 (11.59%)	59 (11.92%)
Alanine aminotransferase(U/L)			
10–40	118 (70.66%)	242 (73.78%)	360 (72.73%)
< 10 or > 40	49 (29.34%)	86 (26.22%)	135 (27.27%)
AST/ALT^a^			
≦1	125 (74.85%)	128 (39.02%)	253 (51.11%)
> 1	42 (25.15%)	200 (60.98%)	242 (48.89%)
Blood urea(mmol/L)^a^			
3.2–7.1	129 (77.25%)	226 (68.90%)	355 (71.72%)
< 3.2 or > 7.1	38 (22.75%)	102 (31.10%)	140 (28.28%)
Blood creatinine(umol/L)			
76–88.41	29 (17.37%)	63 (19.21%)	92 (18.59%)
< 76 or > 88.41	138 (82.63%)	265 (80.79%)	403 (84.41%)
Blood uric acid(umol/L)^a^			
208–428	82 (49.10%)	179 (54.57%)	261 (52.73%)
< 208 or > 428	85 (50.90%)	149 (45.43%)	234 (47.27%)
Erythrocyte sedimentation rate(mm/h)			
≦20	39 (23.35%)	103 (31.40%)	142 (28.69%)
> 20	128 (76.65%)	225 (68.60%)	353 (71.31%)
Total cholesterol(mmol/L)^a^			
2.86–5.98	145 (86.83%)	270 (82.32%)	415 (83.84%)
< 2.86 or > 5.98	22 (13.17%)	58 (17.68%)	80 (16.16%)
Triglyceride(mmol/L)			
0.56–1.7	143 (85.63%)	273 (83.23%)	416 (84.04%)
< 0.56 or > 1.7	24 (14.37%)	55 (16.77%)	79 (15.96%)
High density lipoprotein cholesterol(mmol/L)^a^			
0.94–2	114 (68.26%)	260 (79.27%)	374 (75.56%)
< 0.94 or > 2	53 (31.74%)	68 (20.73%)	121 (24.44%)
Low density lipoprotein cholesterol(mmol/L)			
2.07–3.12	82 (49.10%)	138 (42.07%)	220 (44.44%)
< 2.07 or > 3.12	85 (50.90%)	190 (57.93%)	275 (55.56%)

^a^The parameters were statistically significant by LASSO analysis

*Abbreviations*: *BMI* Body mass index, *ODI* The Oswestry disability index, *JOA* Japanese orthopaedic association scores, *VAS* Visual analogue scale, *ASIA* American spinal injury association, *NCMS* New rural cooperative medical system, *AST* Aspartate aminotransferase, *ALT* Alanine aminotransferase, *HDL-C* High-density lipoprotein cholesterol

**Fig. 1:**
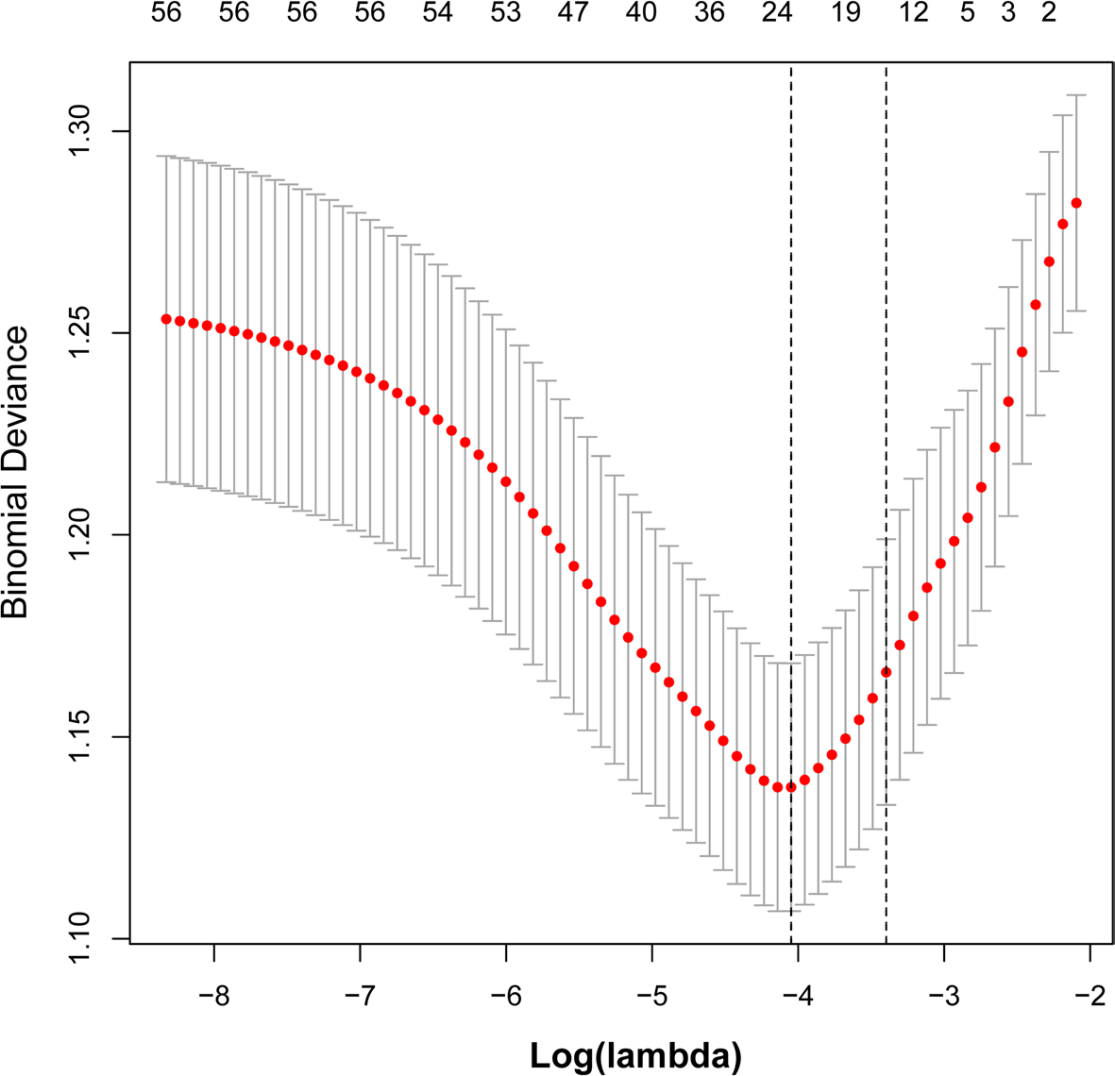
All perioperative parameters were included in LASSO analysis. Binomial deviance was plotted using the LASSO binary logistic regression model, and 24 parameters were statistically significant

**Fig. 2:**
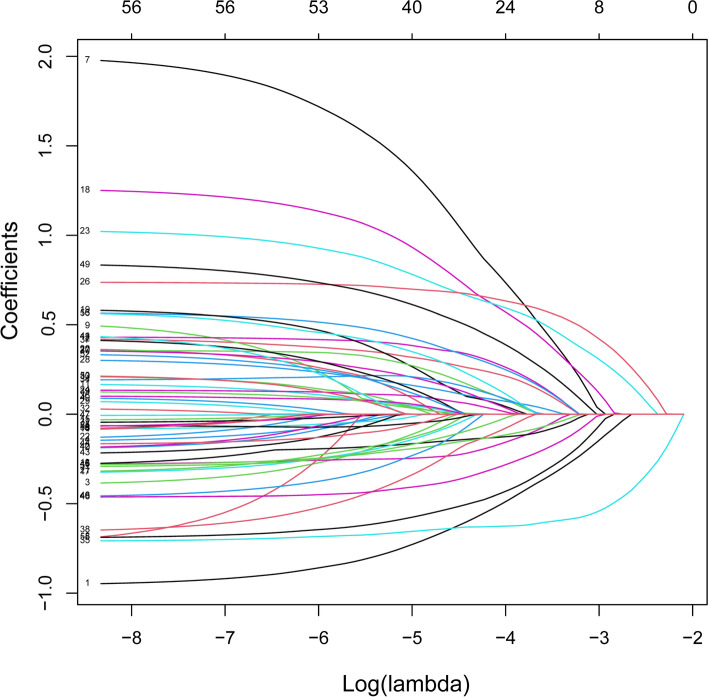
All perioperative parameters were included in LASSO analysis. Coefficient profiles of the 24 features were plotted using the LASSO binary logistic regression model

**Fig. 3:**
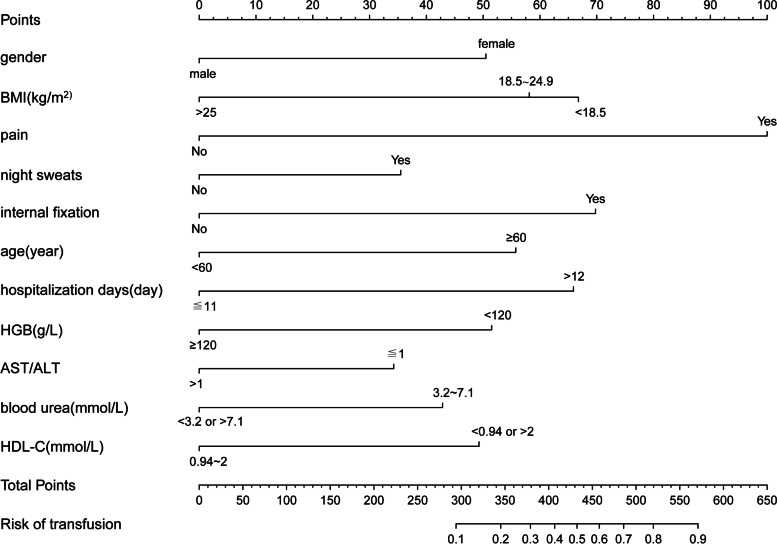
A novel nomogram was constructed to predict blood transfusion risk by calculating the total score of 11 parameters

Data of the two groups were analysed by LASSO regression model analysis, and parameters with statistically significant differences are marked in Table 1. The LASSO regression model analysis identified 24 parameters with statistical differences. These parameters were then analysed by multivariable logistic regression analysis. The parameters with statistically significant differences included sex, body mass index (BMI), pain, night sweats, internal fixation, age, hospitalisation days, HGB, alanine aminotransferase/aspartate aminotransferase (AST/ALT), blood urea, and high-density lipoprotein cholesterol (HDL-C) (Table 2). Binomial deviance (Fig. 1) and coefficient (Fig. 2) were plotted by LASSO analysis. A novel nomogram was constructed to predict blood transfusion risk in spinal tuberculosis surgery (Fig. 3).

**Table 2:** Prediction factors for blood transfusion risk in spinal tuberculosis surgery

Intercept and variable	β	Odds ratio (95% CI)	P值
Gender	−0.8894	0.411(0.251–0.665)	< 0.001*
Course of disease	0.3650	1.441(0.877–2.366)	0.149
BMI1	−0.1503	0.860(0.508–1.463)	0.5769
BMI2	−0.9730	0.378(0.151–0.898)	0.031*
Lower limb pain	6.1821	5.377(1.469–26.835)	0.020*
Night sweats	0.5446	1.724(1.022–2.912)	0.040*
Appetite	−0.1540	0.857(0.537–1.375)	0.520
Focal segmental	0.5344	1.706(0.980–2.973)	0.059
Internal fixation	1.0377	2.823(1.224–7.130)	0.020*
ODI	0.2273	1.255(0.782–2.013)	0.345
Age	0.8349	2.305(1.429–3.745)	< 0.001*
Occupation	0.1275	1.136(0.710–1.824)	0.600
Hospitalization days	0.8010	2.228(1.324–3.780)	0.003*
Surgery classification	0.3840	1.468(0.915–2.373)	0.114
Preoperative hospitalization	0.1957	1.216(0.724–2.033)	0.457
Blood glucose	0.2592	1.296(0.733–2.330)	0.378
White blood cells	0.2748	1.316(0.724–2.442)	0.374
Hemoglobin	−0.6749	0.509(0.313–0.823)	0.006*
Percentage of lymphocytes	−0.3752	0.687(0.428–1.101)	0.119
Albumin	−0.2402	0.786(0.460–1.334)	0.374
AST/ALT	−0.5519	0.576(0.348–0.941)	0.029*
Blood urea	0.7658	2.151(1.288–3.659)	0.004*
Blood uric acid	−0.2588	0.772(0.490–1.214)	0.263
Total cholesterol	0.5102	1.666(0.894–3.192)	0.115
HDL-C	−0.7189	0.487(0.286–0.824)	0.008*

β is the regression coefficient. *: The parameters were statistically significant

*Abbreviations*: *BMI* Body mass index, *ODI* The Oswestry disability index, *AST* aspartate aminotransferase, *ALT* Alanine aminotransferase, *HDL-C* High-density lipoprotein cholesterol

**Fig. 4:**
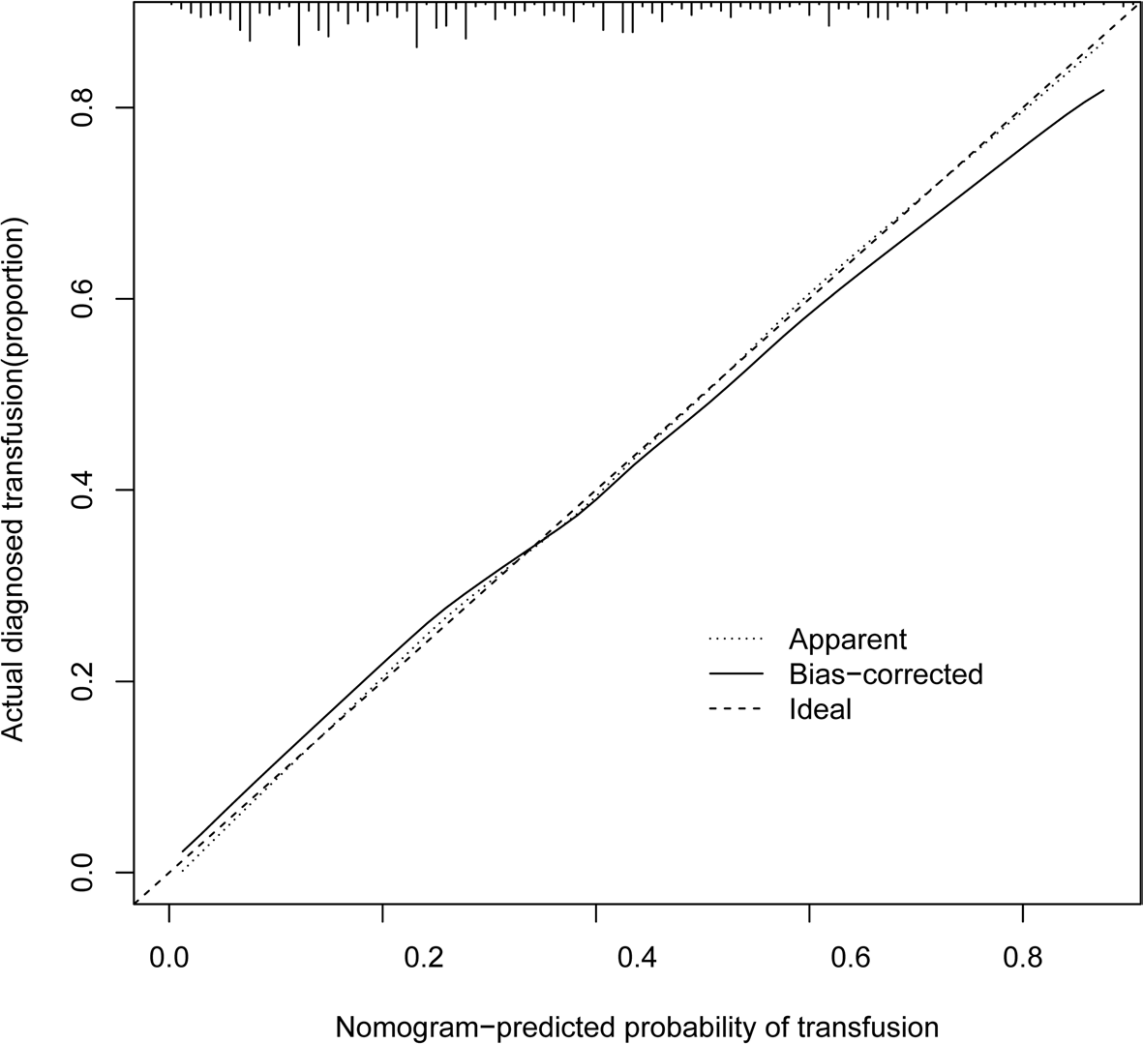
The calibration curve was plotted to exhibit the relationship between the bias-corrected and ideal curve

**Fig. 5:**
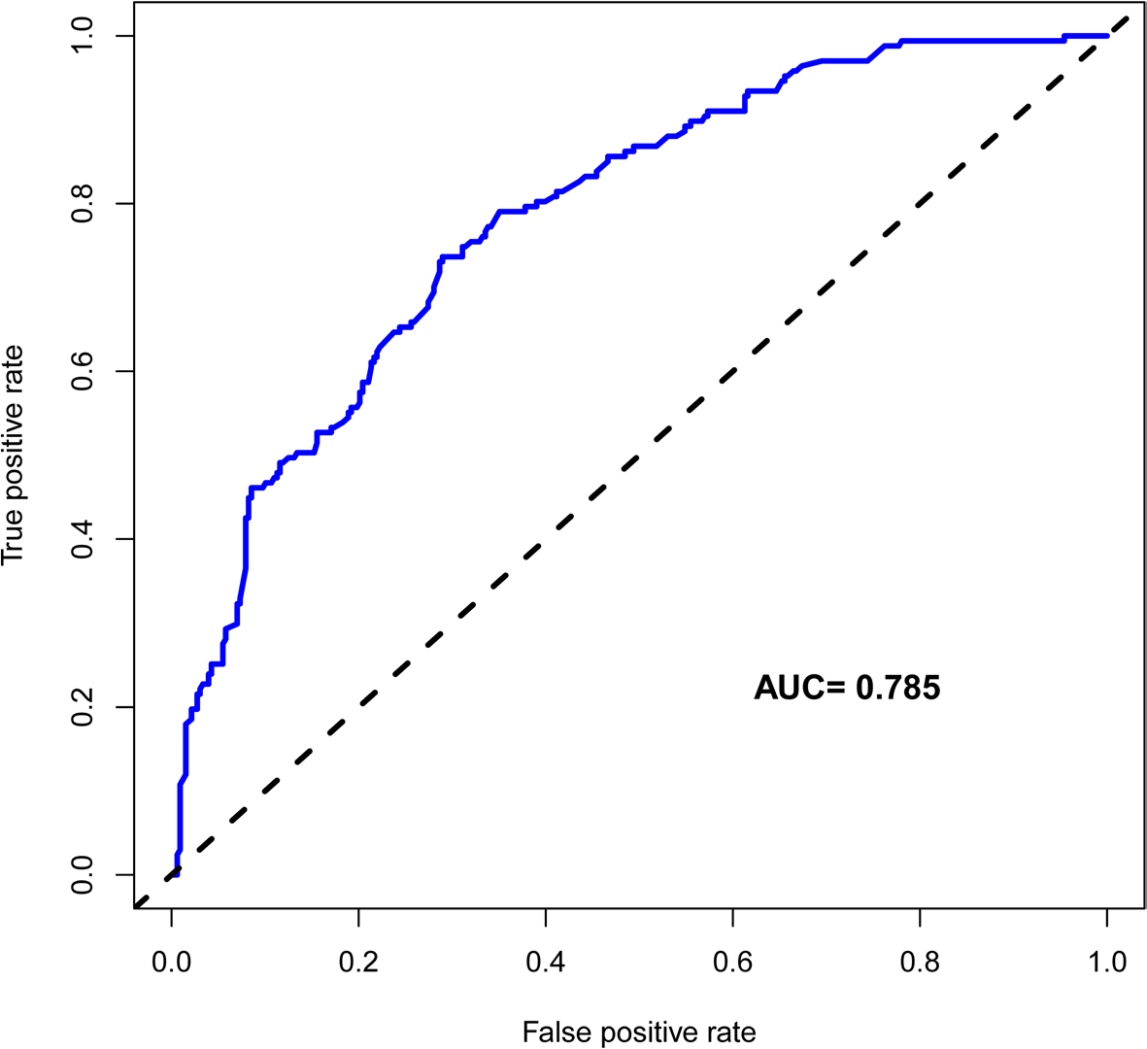
The ROC curve was constructed, and the AUC was 0.785

**Fig. 6:**
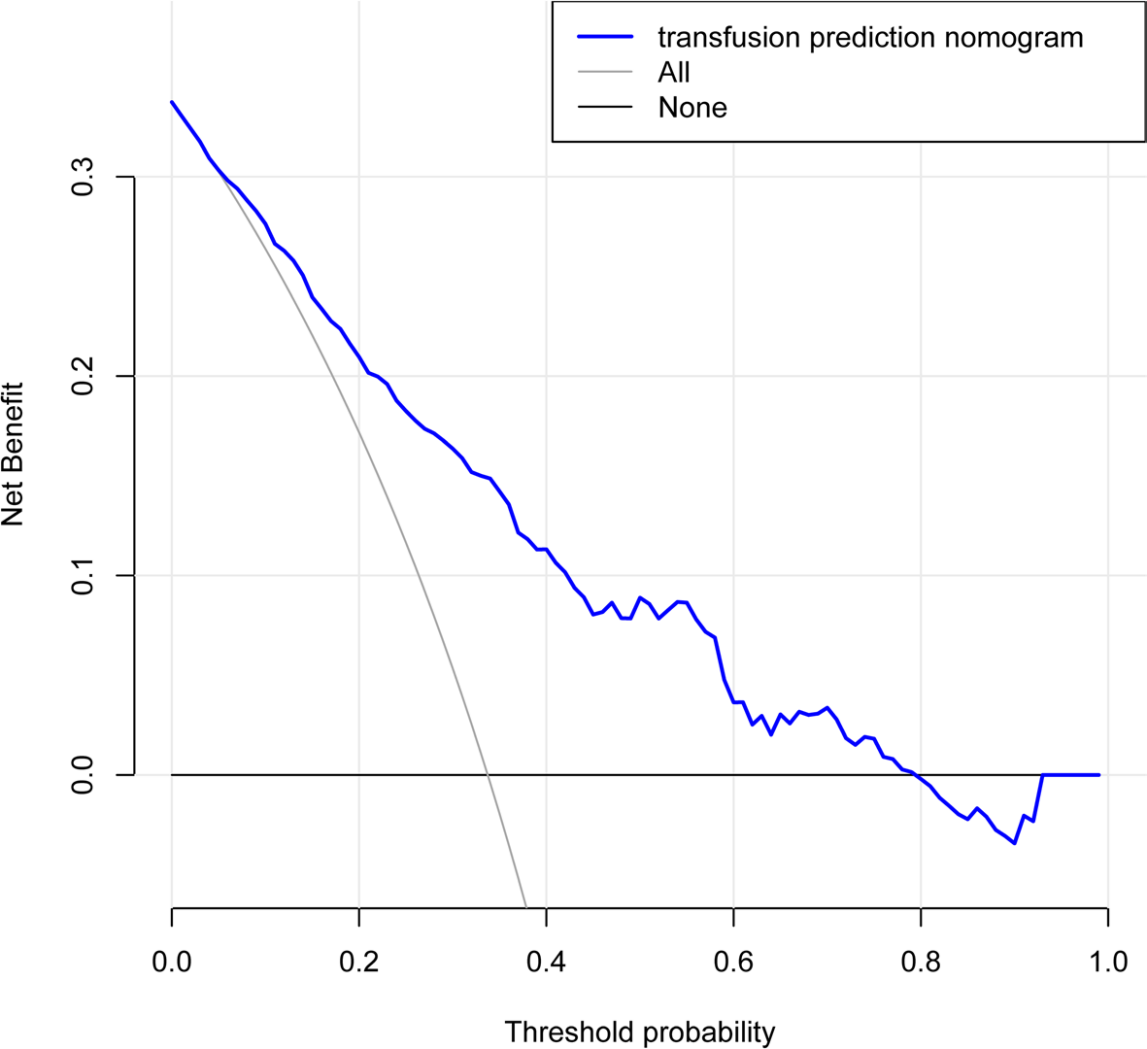
Decision curve analysis for the nomogram of blood transfusion risk. The net benefit of the nomogram was 0.01–0.79

The C-index was measured to evaluate the predictive ability of the new nomogram and was 0.787. The calibration curve was close to the ideal curve, which indicated that the model exhibited superior predictive ability (Fig. 4). The receiver operating characteristic (ROC) curve was constructed, and the AUC was 0.785 (Fig. 5). The net benefit of the predictive nomogram determined by DCA was 0.01–0.79 (Fig. 6).

A relatively corrected C-index was calculated by bootstrapping validation (1000 bootstrap resamples) of the blood transfusion nomogram. The calculated C-index was 0.76, which was close to the C-index of 0.78 measured by the nomogram.

**Fig. 7:**
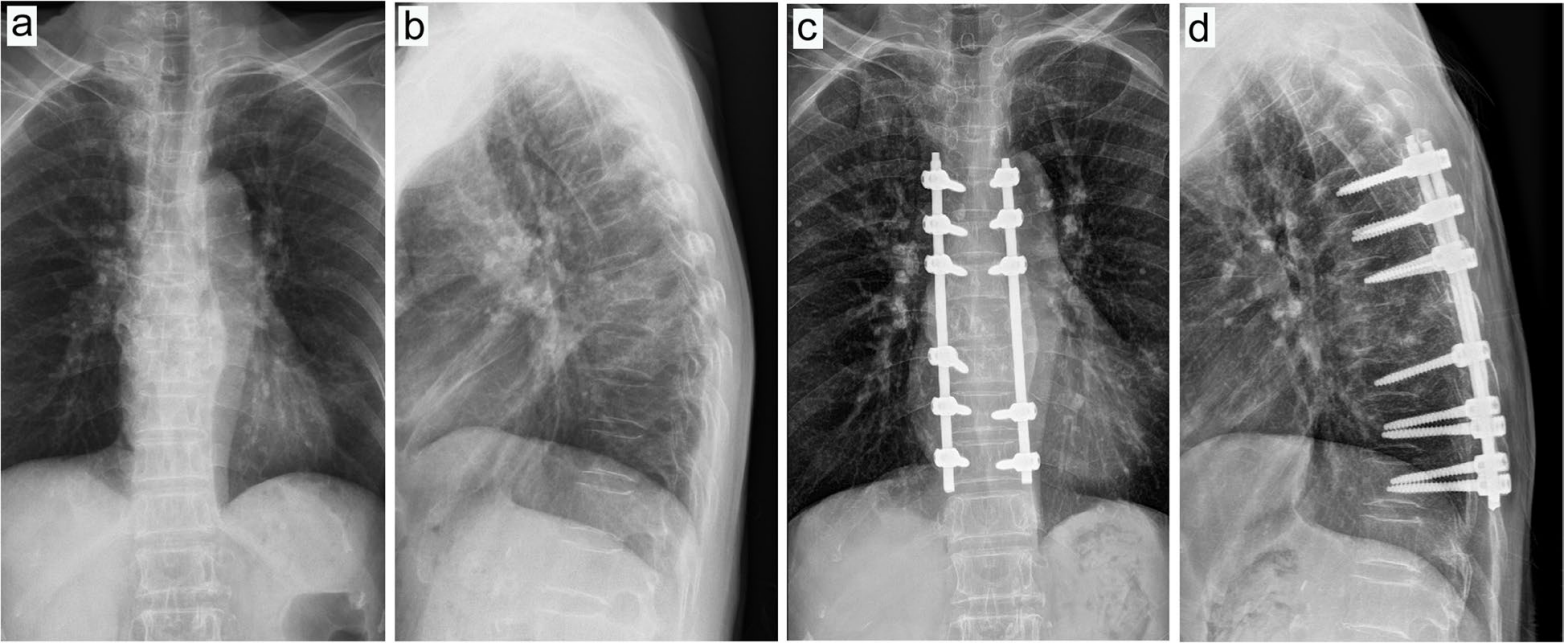
Preoperative and postoperative X-ray examinations in one patient with blood transfusion during surgery. **a**, Preoperative X-ray examination in the positive position. **b**, Preoperative X-ray examination in the lateral position. **c**, Postoperative X-ray examination in the positive position. **d**, Postoperative X-ray examination in the lateral position

**Fig. 8:**
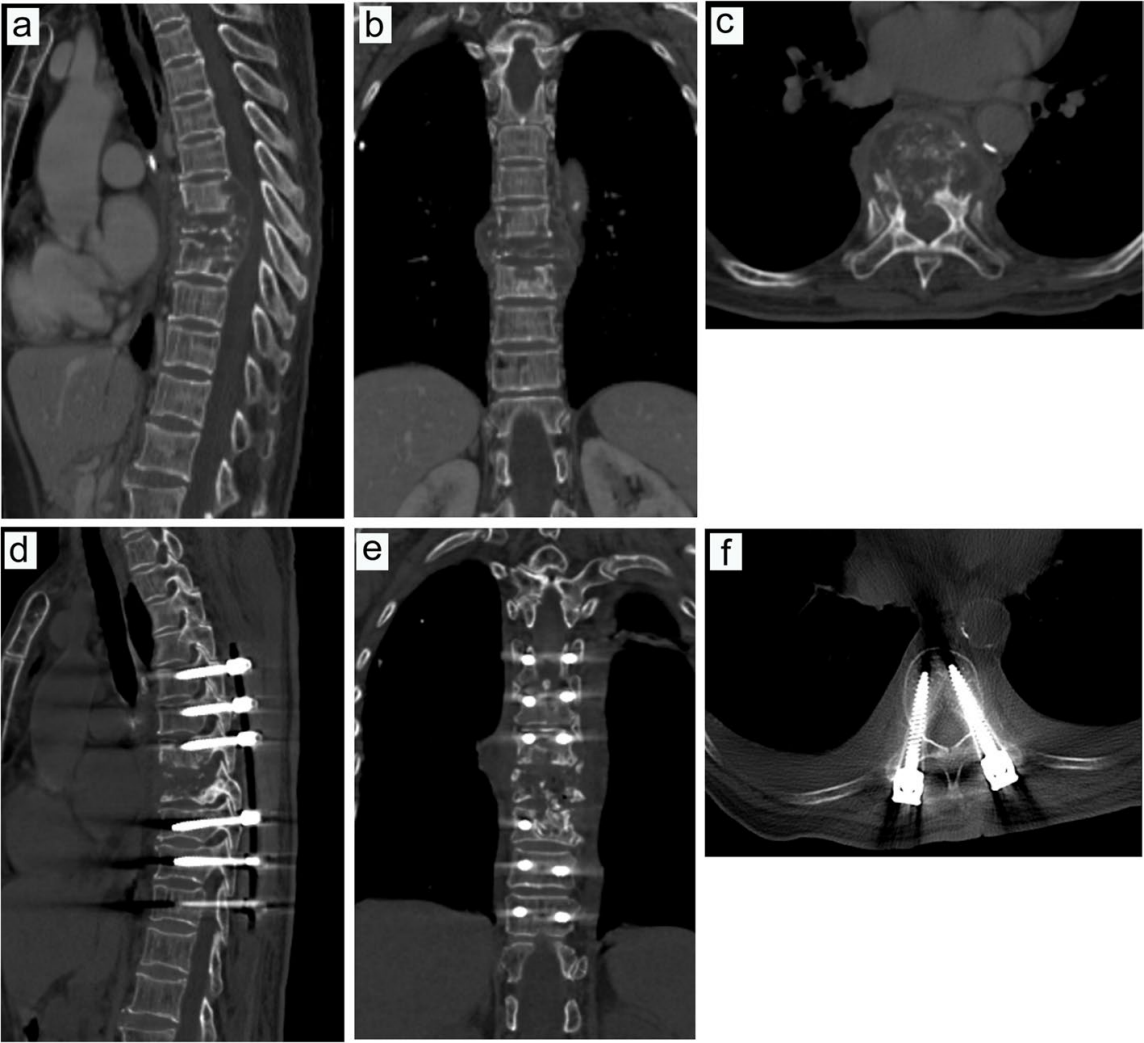
Preoperative and postoperative CT examinations in one patient with intraoperative blood transfusion. **a**, Preoperative CT examination in the sagittal position. **b**, Preoperative CT examination in the coronal position. **c**, Preoperative CT examination in cross-section. **d**, Postoperative CT examination in the sagittal position. **e**, Postoperative CT examination in the coronal position. **f**, Postoperative CT examination in cross-section

**Fig. 9:**
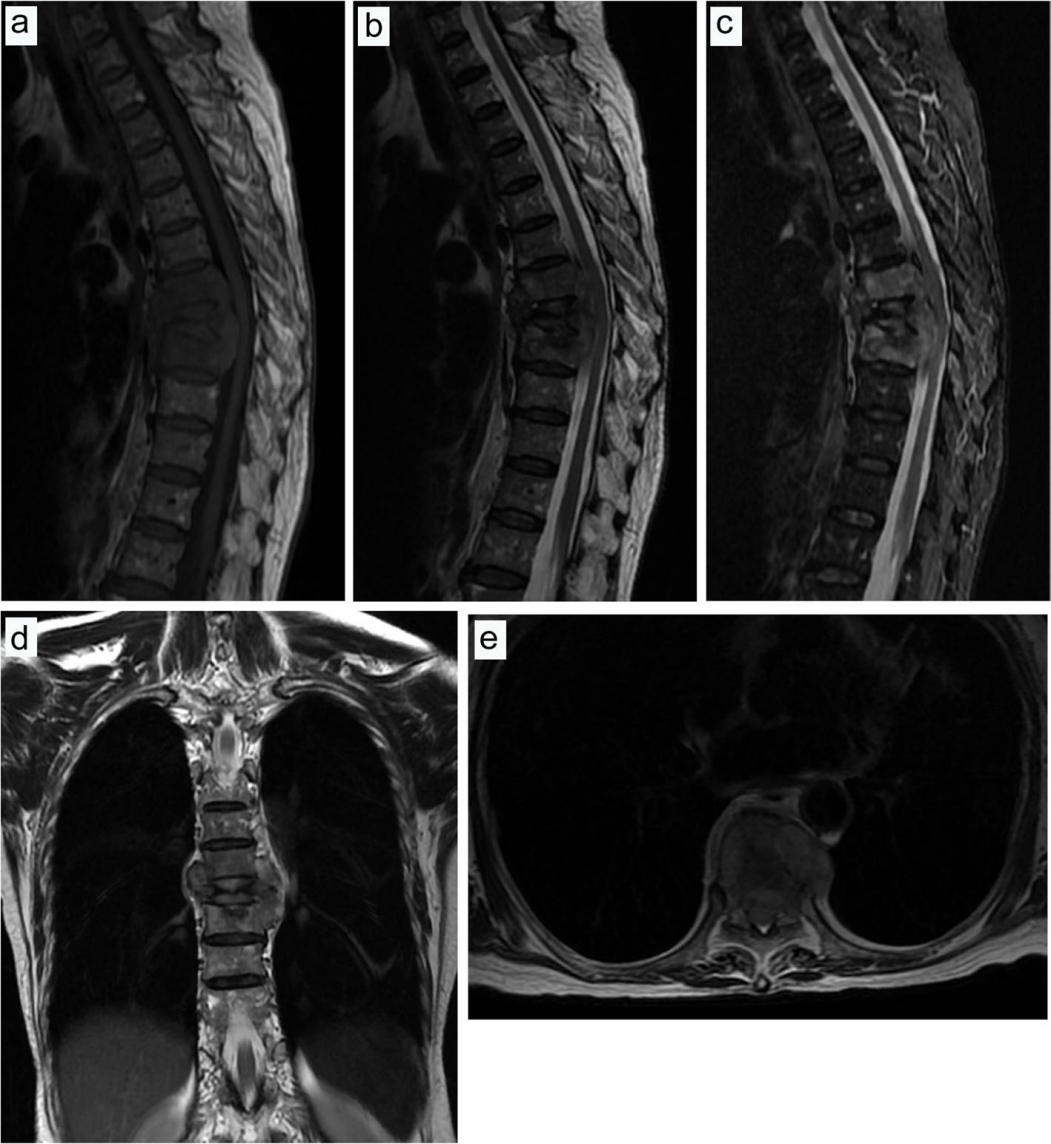
Preoperative MRI examination in one patient with intraoperative blood transfusion. **a**, MRI examination in the sagittal T1 sequence. **b**, MRI examination in the sagittal T2 sequence. **c**, MRI examination of T2 lipid compression sequence in sagittal position. **d**, MRI examination in the coronal T2 sequence. **e**, MRI examination of the T2 sequence in cross-section

**Fig. 10:**
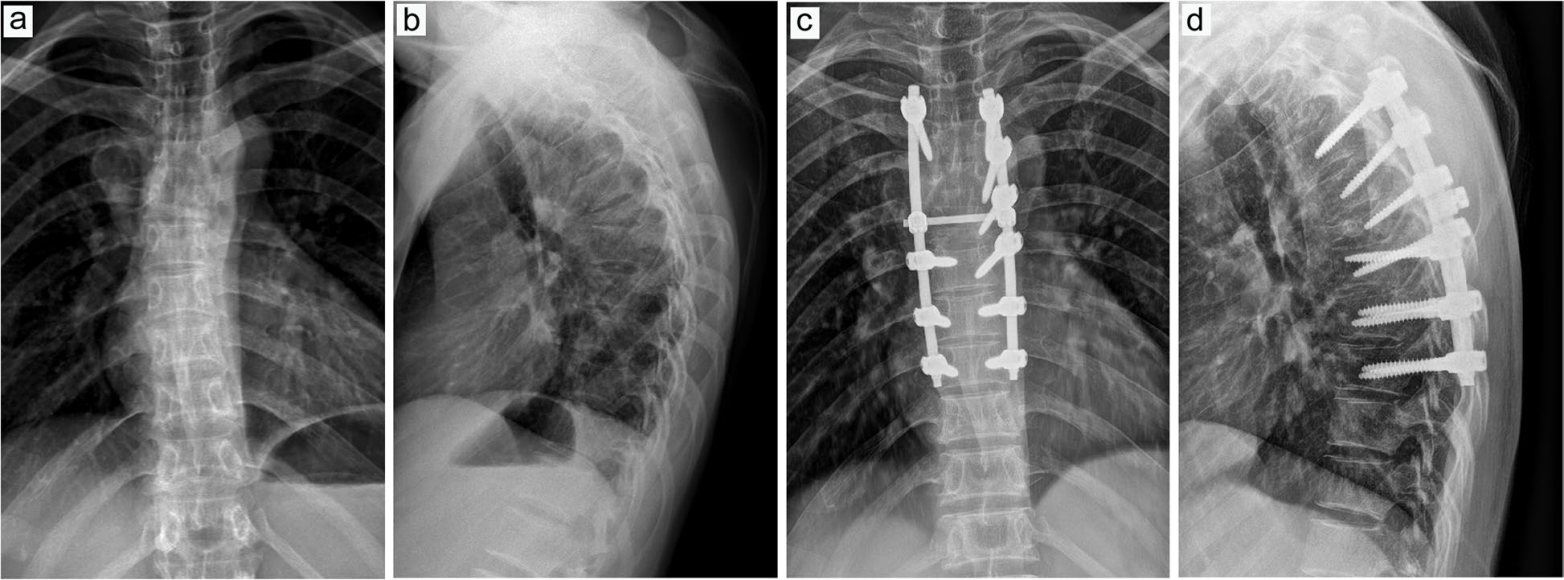
Preoperative and postoperative X-ray examinations in one patient without intraoperative blood transfusion. **a**, Preoperative X-ray examination in the positive position. **b**, Preoperative X-ray examination in the lateral position. **c**, Postoperative X-ray examination in the positive position. **d**, Postoperative X-ray examination in the lateral position

**Fig. 11:**
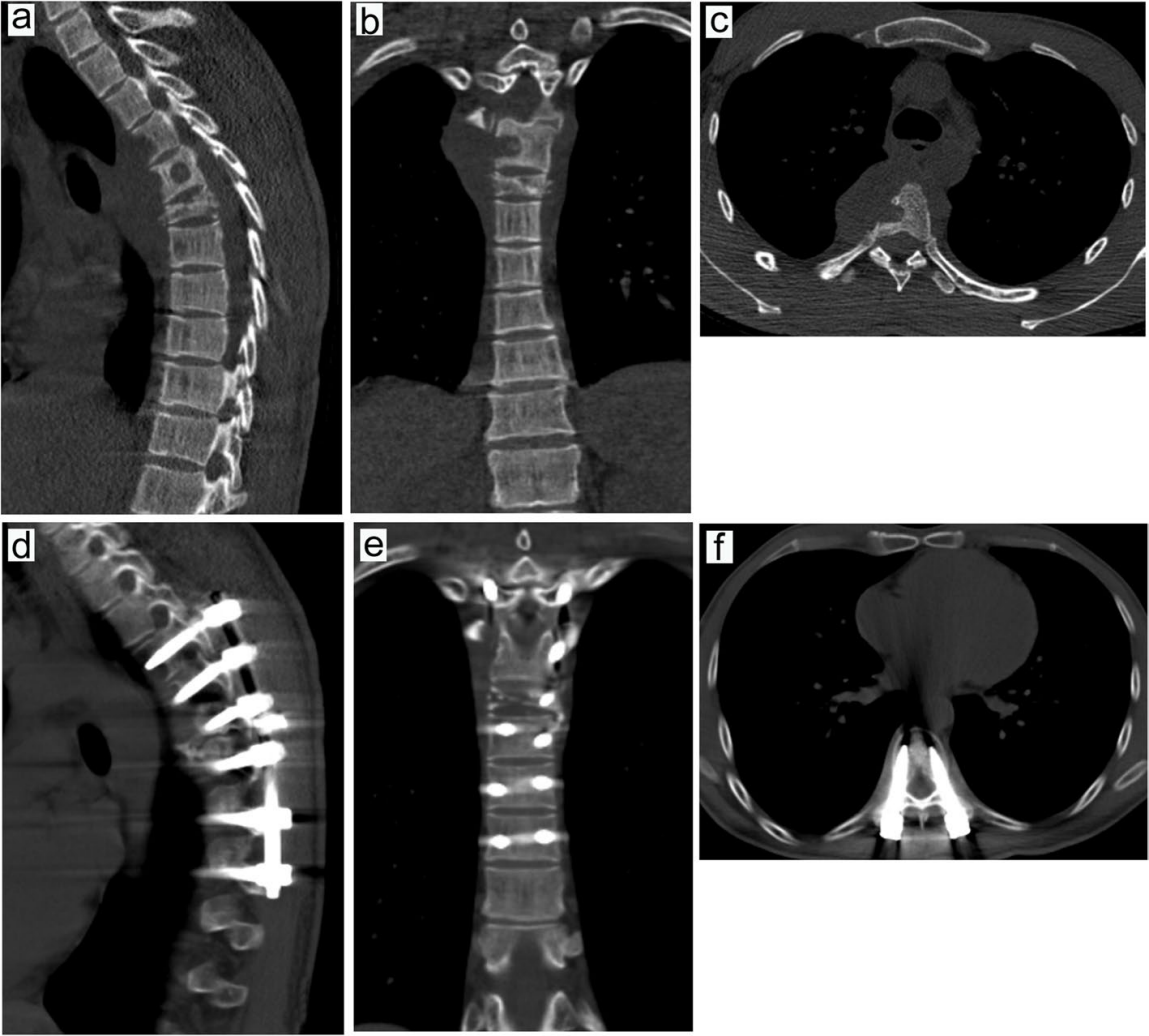
Preoperative and postoperative CT examinations in one patient without intraoperative blood transfusion. **a**, Preoperative CT examination in the sagittal position. **b**, Preoperative CT examination in the coronal position. **c**, Preoperative CT examination in cross-section. **d**, Postoperative CT examination in the sagittal position. **e**, Postoperative CT examination in the coronal position. **f**, Postoperative CT examination in cross-section

**Fig. 12:**
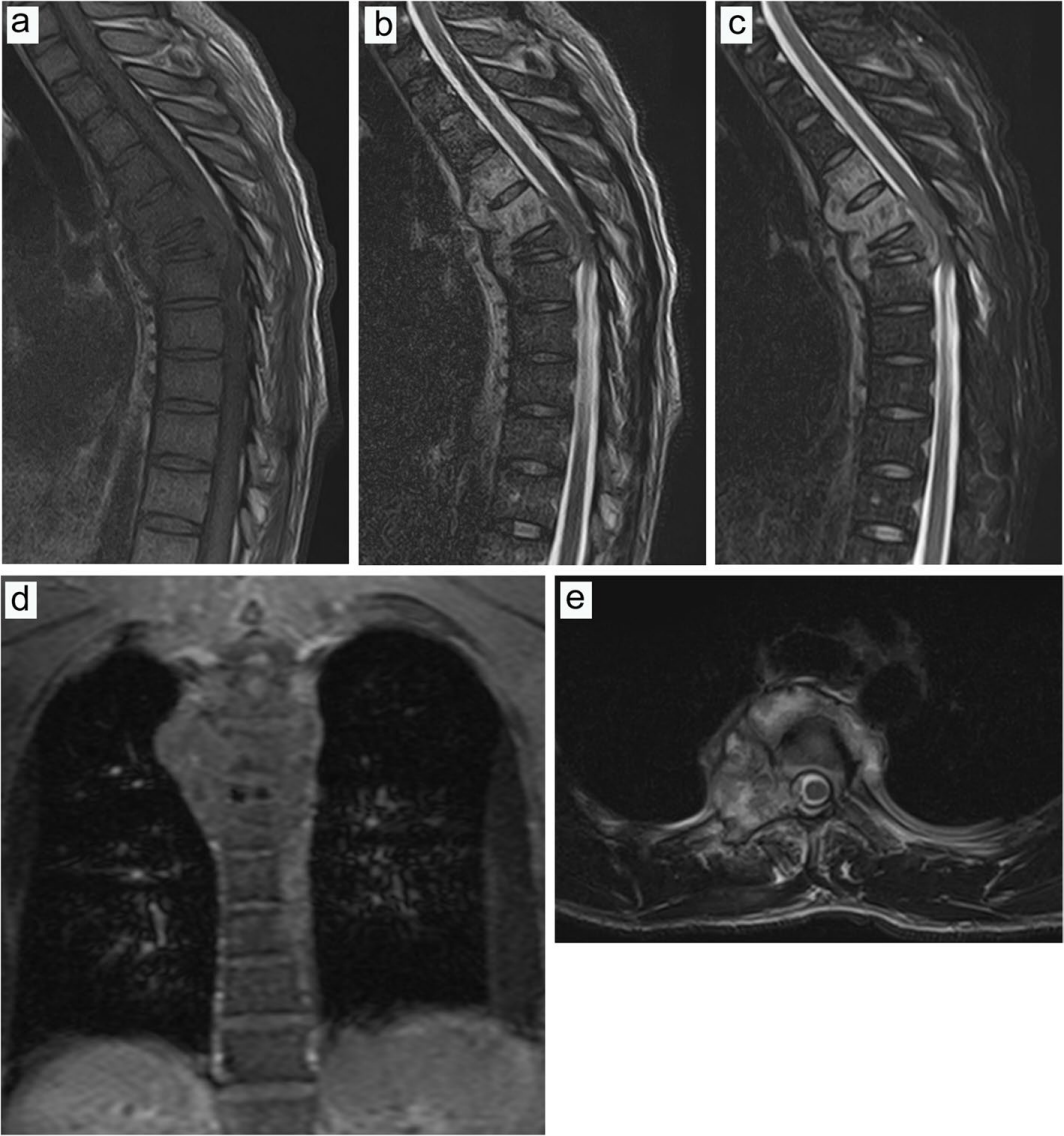
Preoperative MRI examination in one patient without intraoperative blood transfusion. **a**, MRI examination in the sagittal T1 sequence. **b**, MRI examination in the sagittal T2 sequence. **c**, MRI examination of T2 lipid compression sequence in the sagittal position. **d**, MRI examination in the coronal T2 sequence. e, MRI examination of the T2 sequence in cross-section

The present study exhibited two typical cases, comprising one case with blood transfusion (Figs. 7, 8 and 9) and one case without blood transfusion (Figs. 10, 11 and 12).

## Discussion

Typical spinal tuberculosis usually presents with symptoms of tuberculosis poisoning such as pain, fatigue, night sweats, low fever, weight loss, and other symptoms of tuberculosis poisoning [3]. Imaging exhibited bone destruction, and severe tuberculosis of the spine often results in kyphosis [22]. The kyphotic deformity increases surgical difficulty and the risk of bleeding. However, the supply for blood products is inadequate to meet the growing demand for surgery [23]. Therefore, a reasonable prediction of intraoperative blood transfusion in the perioperative period has become crucial.

The present study introduced perioperative parameters to develop the nomogram for predicting the blood transfusion risk. The C-index calculated by the nomogram was 0.787 and 0.763 in the training and validation sets, respectively, which indicated the highly accurate predictability of the nomogram [21]. The C-index calculated in the present paper was higher than that obtained by the previous nomogram (0.734) [24]. The combination and predictive efficacy of the nomogram increased with C-index [21]. The present study used internal validation to calculate the C-index. Studies have shown that good discrimination and calibration ability could be obtained through internal validation in the cohort. The high C-index was especially suitable for wide use in large sample data [21]. The nomogram in the present study achieved the best discriminatory ability with an AUC of 0.785, which was slightly higher than that of the previously established nomogram (AUC = 0.75) [16].

DCA is applied to estimate the clinical usefulness of the nomogram and is a superior tool to estimate the predicted net benefit of the model [25, 26]. Net benefit could be derived based on the threshold probability [27, 28]. The DCA of the nomogram to predict intraoperative red blood cell transfusions could benefit blood transfusions [9].

Blood transfusion risk could be assessed in the perioperative period by using the nomogram model, and the surgeon was well prepared with adequate blood to perform the surgery, which promoted a more reasonable allocation of blood resources [21]. Huang exhibited the efficient development and validation of the nomogram to predict blood transfusion for surgery. This could effectively improve the utilisation of red blood cells for surgery [29]. HGB was crucial to the total score of the nomogram as it contributed up to 50 points to the score. Dominique Engel predicted perioperative blood transfusion in patients undergoing surgery using the same nomogram and exhibited that preoperative HGB was a vital factor affecting blood transfusion [8]. The preoperative low HGB may be closely related to the deficiency of iron, vitamin B12, and folic acid [11, 12].

Female patients were more likely to receive intraoperative blood transfusion than male patients. A study by Stammers involved 54,122 blood transfusion patients and exhibited that the rate of blood transfusion in female patients was almost three times higher than that in male patients, [13]. This finding is concurrent with that of Cao et al. [14]. This may due to the application of the same absolute transfusion strategy by clinicians and the performance of liberal transfusion strategy in clinical settings [15].

In the present study, internal fixation was a predictor of blood transfusion during spinal tuberculosis surgery. The increase in the surgical exposure range as the number of pedicle screws implanted leads to an increase in surgical bleeding. The study by Ding et al. found that the mean difference of incision length of fenestration discectomy was 3.74 cm longer than that of percutaneous transforaminal endoscopic discectomy, and the mean difference of amount of bleeding was 63.66 mL higher than that of the latter [30]. In addition, the vertebrae are rich in blood, and blood flows out of the orifice when the pedicle is drilled and the internal fixation device is inserted. The haemorrhage increases with the number of pedicle screws implanted during surgery, making intraoperative blood transfusion necessary. Yang et al. observed that surgical bleeding increased with an increase in the number of internal fixations [31]. Shi et al. observed that the bleeding volume of internal fixation during spinal tuberculosis surgery ranged from 467.7 to 2833.3 mL [32].

Age was a predictor of blood transfusion during spinal tuberculosis surgery in the present study, with elderly patients exhibiting a higher frequency of transfusion. The fragility of blood vessels in elderly patients increased, and the coagulation factor activity in these patients was lower than that in young patients, resulting in increased surgical bleeding. Nie et al. included 565 elderly people to construct a blood transfusion prediction model for spinal surgery and observed that age was a crucial influencing factor [33]. These findings were concurrent with those of Liu et al. [34]. Additionally, the present study exhibited that lower BMI was a predictor of the risk of spinal tuberculosis blood transfusion. These results were similar to those of Liu et al., who exhibited that although the BMI decreased from 44 to 14, the ability to predict the risk of blood transfusion gradually increased [34].

Although the association between analgesics and surgical bleeding has been rarely explored, the model constructed in the present study suggested that pain was a predictor of spinal tuberculosis surgical blood transfusion. It has been reported in the literature that spinal pain is closely related to inflammatory factors [35]. Bacteria infect surrounding tissues leading to inflammatory edema, increasing vasculitis permeability and necrotic vasculitis [36]. The study by Oehlers et al. showed that inflammatory granuloma induced an increase in vascular permeability and thus contributed to the spread of *Mycobacterium tuberculosis* [37]. Therefore, patients with inflammatory pain tend to increase the amount of intraoperative blood loss.

The present study has some limitations. This article was a retrospective study and lacked validation from prospective studies. Additionally, the study spanned 10 years, and the results were influenced by changes in surgical procedures. A larger sample size is required to further validate the efficacy of the nomogram.

## Conclusions

A nomogram with high accuracy, clinical validity, and reliability was established to predict blood transfusion risk in spinal tuberculosis surgery. The perioperative factors that predicted blood transfusion were sex, BMI, pain, night sweats, internal fixation, age, hospitalisation days, HGB, AST/ALT, blood urea, and HDL-C. This nomogram had good clinical validity and reliability. Surgeons must prepare preoperative surgical strategies and ensure the availability of adequate blood before surgery. Use of the nomogram to predict blood transfusion risk in spinal tuberculosis surgery may ensure appropriate utilisation and distribution of blood products.

## Data Availability

Data sharing is not applicable to this article as no datasets were generated or analyzed during the current study.
